# A Unique Case of Cardiac Hypereosinophilia With Recurrent Valve Thrombosis After Mechanical Mitral Valve Replacement in the Setting of a Supratherapeutic International Normalized Ratio

**DOI:** 10.7759/cureus.25301

**Published:** 2022-05-24

**Authors:** Maja Ostojic, Brendan J Carry, Ankit Kumar, Kathie Wu

**Affiliations:** 1 Internal Medicine, Geisinger Medical Center, Danville, USA; 2 Cardiology, Geisinger Health System, Danville, USA

**Keywords:** mechanical valve thrombosis, supra-therapeutic inr recurrent valve thrombosis, mechanical mitral valve replacement, cardiac hypereosinophilia, hypereosinophilia syndrome

## Abstract

Hypereosinophilia (HES) is a rare, but highly fatal, disease that results in excess eosinophils causing multiorgan damage, mainly manifesting as extensive inflammation contributing to fibrosis. Notably, cardiac involvement occurs in almost half the cases and can often lead to thrombus development. This is a unique case of HES contributing to recurrent cardiac thrombus formation on a mechanical mitral valve in the setting of a patient who had a supratherapeutic international normalized ratio (INR) while on coumadin. The rarity of this case is also displayed in the patient’s negativity for one of the fusion genes that are highly suggestive of cardiac HES, the demographics of her female gender, and her first objective sign being T-wave inversions versus the usual heart failure signs and symptoms. This case raises awareness of the disorder but also the importance of keeping its potential exacerbations on the differential, even in the setting of atypical presentations. With this, it also begs the question of whether coumadin is the proper anticoagulant of choice in these patients and whether other parameters should be considered.

## Introduction

Hypereosinophilia (HES) is a multisystem disease with a poor prognosis and an average prevalence of 3% [1]. Eosinophils are normally present in the blood in small numbers with the count usually being less than 350/mm^3^. Hypereosinophilia is therefore defined by an eosinophil count of > 1.5 x 109/L on two tests greater than one month apart or tissue hypereosinophilia plus organ damage directly caused by the HES [1]. Pathophysiologically, the proposed mechanism is twofold: 1) clonal eosinophilic proliferation as a result of a primary molecular defect involving hematopoietic cells and/or defects in signal transduction from the receptors that mediate eosinophil production, and 2) overproduction of eosinophil cytokines such as interleukin 5 (IL-5) [2-3].

Demographically, HES has a 9:1 predilection for males, and it affects patients in the 20-50 age range [3]. The mortality rate is approximately 75% in untreated patients three years after diagnosis [4]. The symptoms at initial presentation are as follows: dermatologic (37% of the time), pulmonary (25%), gastrointestinal (14%), cardiac (5%), and neurologic (4%) [4-5].

Approximately 40% of the time, there is some sort of cardiac involvement later on in the disease course [4]. The severity of the cardiac injury doesn’t clearly correlate with the degree of peripheral eosinophilia, but there is a high association with the FIP1L1-PDGFR⍺ fusion gene. One of the only available therapies for this disease, imatinib mesylate, acts directly on that gene [6].

The cardiac involvement consists of (1) the necrotic stage, (2) the thrombotic stage, and (3) the fibrotic stage. Essentially, cytokines are released by eosinophils to initiate coagulation, activating platelets and inhibiting anticoagulant activity, leading to thrombin formation and clot generation. The clotting potential of eosinophils in this disorder is, therefore, high and intracardiac thrombi are common thromboembolic sequelae of the disease. In fact, almost 25% of HES patients have some type of clot complication and 5-10% die of them such as the unfortunate case of our patient. Aside from heparin or low-molecular-weight heparin being used for patients in the hospital, coumadin has been the mainstay of outpatient anticoagulation therapy in these patients. The duration of treatment has often been determined by the degree of the patients’ endomyocardial disease, but set guidelines have not been established in regards to duration or titration parameters [7].

## Case presentation

A 57-year-old female presented with symptoms of shortness of breath, orthopnea, dry cough, and fever. Her past medical history was significant for hypereosinophilia (diagnosed nine years prior), endocardial fibroelastosis, atrioventricular nodal reentry tachycardia status post-ablation, left ventricular thrombus (on chronic coumadin), severe mitral valve regurgitation status post mechanical valve replacement, atrial fibrillation/flutter status post two cardioversions, heart failure with preserved ejection fraction, and moderate to severe pulmonary hypertension.

Just 15 days prior to the current presentation, the patient was admitted for sepsis. Her course was complicated by a heart failure exacerbation and she was sent home on 40 milligrams of Lasix twice a day as well as two liters of oxygen with ambulation and one liter at night. An echocardiogram was done at that time, which showed moderate tricuspid regurgitation, severe dilation of her left atrium, and a mitral valve bileaflet disc with no stenosis or vegetations.

Upon discharge, the patient did well for about a week, however, her oxygen requirement later increased to five liters throughout the day during this next acute presentation. On physical exam, she was normotensive with borderline tachycardia, had elevated jugular venous pressure, fine crackles bilaterally with diminished lung sounds at the bases, as well as trace bilateral lower extremity edema. Her labs showed an elevated international normalized ratio (INR) of 3.89, pro-brain natriuretic peptide (pro-BNP) of 11,835 (increased from 2,000 15 days prior), leukocytosis of 37,760, as well as positive esterase and nitrites in her urine. Her electrocardiogram and a chest X-ray were insignificant and unchanged from earlier. Due to appearing volume-overloaded, the patient was diuresed more aggressively. Aside from a heart failure exacerbation, alternative differentials included worsening of her underlying HES, superimposed infection, drug toxicity (with the amiodarone she was using for atrial fibrillation/flutter), and diffuse alveolar hemorrhage (DAH) (given that she had the risk factor of being on coumadin).

The following day, she became hypoxic, requiring 60 liters of high-flow nasal cannula. She showed no clinical improvement after a trial of diuresis and was transferred to the critical care service. The patient became febrile overnight and the hypothesis was that the increased metabolic rate from the fevers was possibly driving the hypoxia. A CT chest with contrast showed concern for opportunistic infection versus pneumonitis with worsening ground-glass opacities. An infectious workup was initiated, revealing only a positive urine culture with Citrobacter freundii. The patient was started on broad-spectrum antibiotics. She developed a 1-gram drop in her hemoglobin with no obvious signs of bleeding, so DAH remained on the differential in the setting of her chronic anticoagulation with the presenting supratherapeutic INR.

The next day, the patient's oxygenation continued to worsen and a repeat chest X-ray showed a significant worsening of her diffuse bilateral infiltrates. The patient was subsequently intubated and a bronchoscopy was performed with no gross abnormalities noted. Infectious disease was consulted, who recommended stopping the antibiotics in lieu of a negative infectious workup and an asymptomatic urinary tract infection.

The patient subsequently developed a narrow pulse pressure that was more consistent with a heart failure hypovolemic etiology. A CT-pulmonary embolism study showed increasing diffuse ground-glass opacities and patchy alveolar consolidations, suggestive of a worsening infectious/inflammatory process. Hematology/oncology was consulted due to the leukocytosis now being 82,960. Since the patient was on high-dose steroids during this hospitalization, that was deemed the likely culprit. HES versus amiodarone-induced lung toxicity rose higher on the differential.

**Figure 1:**
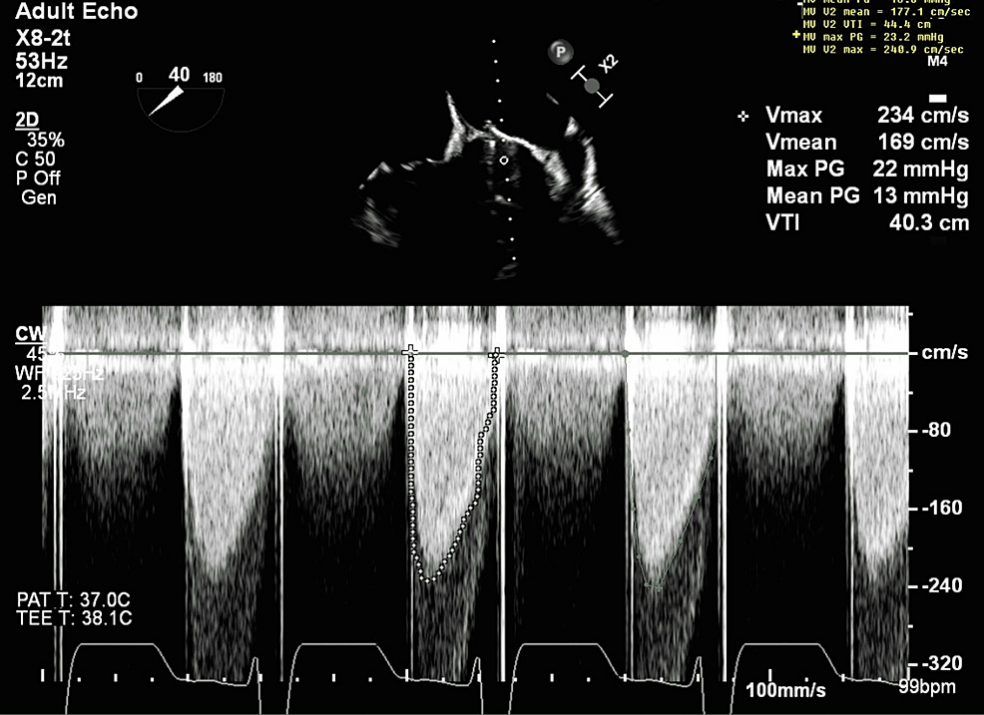
Elevated transmitral diastolic pressure gradient

**Figure 2:**
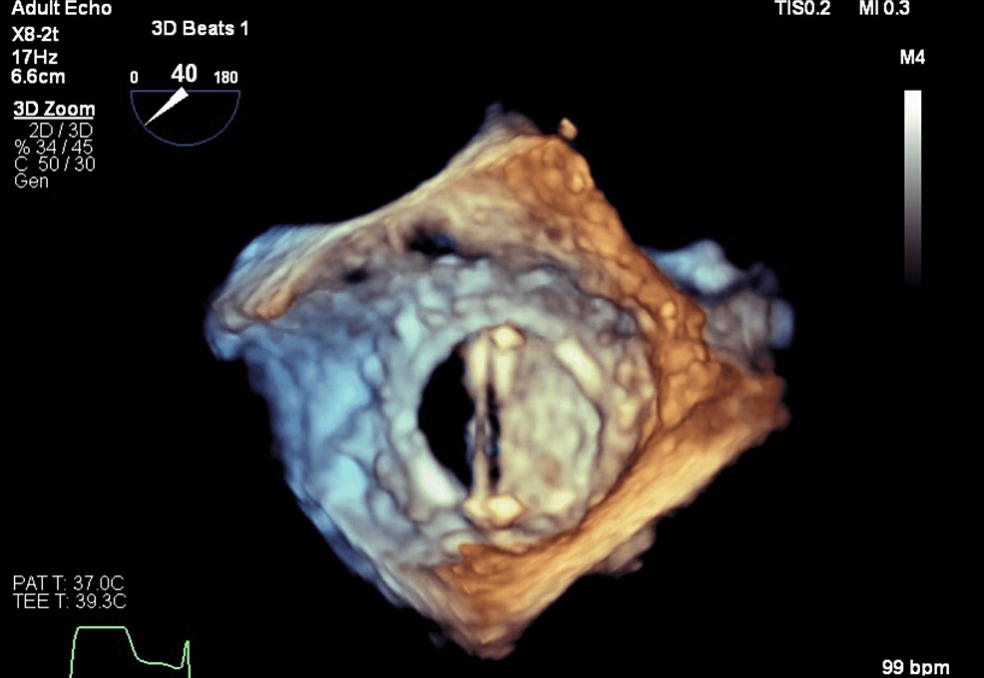
Mechanical bileaflet prosthesis with one immobile leaflet

A formal echocardiogram was done, which showed an increased right ventricular dilation/dysfunction with severe mitral regurgitation and raised pulmonary pressures (Figure 1). The patient was taken to the cath lab for a fluoroscopic view of the valve, which demonstrated a bileaflet mitral valve with one leaflet immobile and the other showing minimal movement (Figure 2). An acute cardiac thrombotic event rose to the top of the differential. The patient was therefore thought to be in cardiogenic shock with gradients across the mechanical valve being markedly elevated (13.8 mmHg), suggesting a severe obstruction. The patient was cannulated for veno-arterial (VA) extracorporeal membrane oxygenation (ECMO) by cardiothoracic surgery and transferred to the cardiac intensive care unit. Despite efforts, her creatinine doubled and she became anuric with hyperkalemia, requiring a nephrology consult and continuous renal replacement therapy (CRRT) initiation. She became acutely anemic, requiring a transfusion, developed a shock liver state, and required Rasburicase for elevated uric acid levels.

A goals-of-care discussion was held with the patient and her family. It was theorized that she likely thrombosed her valve again given her complex hematologic disorder. Her blood cultures remained negative and the transesophageal echocardiogram (TEE) showed that the valve was functioning normally just two weeks prior. The pathology from her previous valve surgery suggested the possibility of “chronic endocarditis” but no organism was ever isolated. The patient and family expressed that they did not want to go through another heart surgery (even if life-saving) and that they only wanted to attempt less invasive measures to resolve the valve thrombosis. A tissue plasminogen activator (tPA) was therefore given. A repeat transthoracic echocardiogram (TTE) showed improvement in the mitral valve mean gradient from 19 mmHg to 9 mmHg and tPA was repeated the following day. A repeat TEE revealed an immobile leaflet of the mitral valve prosthesis, one of them now being free. There was no apparent residual obstructing debris and the consensus opinion was that no further thrombolysis would be beneficial. After further discussion with the family, the decision to decannulate the ECMO was made. This resulted in terminal extubation and patient expiration.

## Discussion

Cardiac HES presents with a variety of cardiac signs and symptoms. In order of prevalence, they are: signs of heart failure (75%), dyspnea on exertion (60%), endocardial fibrosis/myocardial inflammation/mural thrombus formation (57%), mitral regurgitation murmur (49%), cardiomegaly (37%), and T-wave inversions (37%) [4]. There are specific echocardiographic findings that point towards the diagnosis, which include: 1) obliteration of the apex of the left ventricle or right ventricle (or both) by a laminar thrombus (due to more static blood flow there), 2) myocardial thickening, and 3) atrioventricular valvular regurgitation (due to chordae tendinae entrapment by the thrombus) [7]. Recent studies have shown that MRI is the best tool to detect even the first (necrotic) stage of the disease. Endomyocardial biopsy is the gold standard of definitive disease but is reserved for patients where there is uncertainty about the cause of cardiac disease [8].

Furthermore, the cardiac manifestations of HES are broken down into three main stages. The first stage is the necrosis stage, which occurs by about 5.5 weeks of disease onset. It is characterized by infiltration of the endocardium and myocardium by eosinophils, resulting in their degranulation and the formation of sterile microabscesses. These microabscesses are usually asymptomatic and not identified on any imaging modalities this early on. The second stage is the thrombotic stage, which is characterized by thrombi formation on the endocardial surface of the heart and is often complicated by embolic phenomena. After a mean HES duration of at least 10 months, there is high cardiac thrombus mobility and dislodgement potential. The third stage is the fibrotic stage (formerly called Loeffler’s endocarditis), which has a mean duration of disease onset of about 24.5 months. This stage is characterized by collagen replacing the thrombi and causing fibrosis, which leads to restrictive valve motion and regurgitation, as well as restrictive cardiomyopathy and heart failure [7].

The incidence of mechanical valve thrombosis is about 0.6-6% for left-sided heart valves and up to 20% for right-sided heart valves [9]. The most common cause or risk factor for valve thrombosis is inadequate anticoagulation therapy. The target INR for the mechanical mitral valve is 2.5-3.5. There have been very few recorded cases of not only thrombosis but even re-thrombosis of the mechanical valve, especially in the setting of a supratherapeutic INR such as seen in our patient. Her presenting INR was 3.89 and prior to the heparin bolus that she received during cannulation, her INR was almost doubled at an unexplained 6.11. Currently, the mainstay of therapy for mechanical valve replacement (even in HES) is coumadin. It is unknown exactly why this re-thrombosis occurred in our patient, and it certainly begs the question of whether coumadin is truly the appropriate medication of choice in this niche patient population. There have been very limited studies on this particular question though. One idea that has been proposed is to perhaps use the prothrombin time (PT) rather than the INR, a more reproducible measure [7].

Another aspect of uniqueness, in this case, is that our patient is in the demographic minority of being a female in this heavily skewed 1:9 female to male ratio of patients affected by HES. Additionally, not only did she have initial cardiac involvement, which only occurs in 5% of patients, but she tested negative for the FIP1L1-PDGFR⍺ fusion gene, which is highly associated with cardiac injury in hypereosinophilia. Another layer of rarity also exists in the fact that upon initial presentation nine years ago, she did not come in with symptoms of heart failure or shortness of breath but rather T-wave inversions as the first noted sign. This was seen on a preoperative clearance EKG when the patient was being cleared for a tibialis tendon tear repair, and the T-wave inversion was a new finding compared to an EKG done the year prior. The surgery was therefore canceled for further workup, and the patient actually ended up hospitalized due to new-onset chest pain. At that time, endocardial fibroelastosis was discovered in the cardiac CT. An MRI was further done, which showed mixed inflammatory infiltrates and a chronic clot with no acute clot found - a clot that throughout the next few years disappeared without treatment and then reappeared again.

Even with her unfortunate expiration, our patient was in the 25% minority of living past the three-year mortality mark after the initial diagnosis.

## Conclusions

HES is a complicated systemic disorder with a high mortality rate. Its high incidence of clotting and fibrosis of the heart valves predisposes patients to cardiac thrombus formation. Coumadin is the drug of choice in patients who undergo mechanical valve replacement, with or without HES. There have not been specific studies in this patient population, and perhaps another agent (or evaluation of another parameter such as PT) would better serve to prevent thrombus formation and even re-thrombosis such as seen in our patient. This case, therefore, raises the need for further research in regard to anticoagulation in patients with HES and cardiac involvement.

Additionally, it emphasizes the importance of early fluoroscopy and early TTE/TEE imaging in this patient population with each readmission. Even though their presentation may be atypical for thrombus formation, dangerous changes can occur even as little as two weeks, as seen in our patient. An acute on chronic HES exacerbation should always be high on the differential in these patients, and early detection is imperative.
